# Trisomy Chromosome 6 as a Sole Cytogenetic Abnormality in Acute Myeloid Leukemia

**DOI:** 10.4274/tjh.2013.0107

**Published:** 2015-02-15

**Authors:** Monika Gupta, Nita Radhakrishnan, Manoranjan Mahapatra, Renu Saxena

**Affiliations:** 1 All India Institute of Medical Sciences, Department of Hematology, New Delhi, India

**Keywords:** Acute myeloid leukemia, Trisomy 6, cytogenetics

## Abstract

Identification of cytogenetic abnormalities plays an important role in the diagnosis and prognosis of leukemias. Isolated trisomy 6 is a rare abnormality, the prognostic significance of which is not well established. We report one case of acute myeloid leukemia (AML-M5 variant) with trisomy 6 as the sole cytogenetic abnormality. Previously, trisomy 6 has been reported in aplastic anemia, myelodysplastic syndrome, and AML, usually associated with hypocellular marrow. However, our patient had a very short history and hypercellular marrow infiltrated with blasts. We report this case due to the rarity of the condition. More studies are required to ascertain the role of trisomy 6 in the development of leukemia as well as in prognosis.

## Abstract (tr)

Sitogenetik anormalliklerin tanımlanması lösemilerin tanı ve prognozunda önemli bir rol oynar. İzole trizomi 6, prognostik önemi iyi belirlenmemiş olan nadir bir anormalliktir. Tek sitogenetik anormallik olarak trizomi 6’ya sahip bir akut miyeloid lösemi (AML-M5 varyantı) olgusunu sunuyoruz. Trizomi 6 daha önce aplastik anemi, miyelodisplastik sendrom ve genellikle hiposelüler ilik ile ilişkilendirilmiş AML’de bildirilmiştir. Ancak hastamızın çok kısa bir geçmişi ve blastlar ile infiltre hiperselüler bir iliği vardı. Bu durumun nadirliği nedeni ile bu olguyu rapor ediyoruz. Hem lösemi gelişiminde trizomi 6’nın rolünü tespit etmek, hem de prognoz için daha fazla çalışma gereklidir.

## INTRODUCTION

Identification of cytogenetic abnormalities plays an important role in the diagnosis and prognosis of leukemias. However, apart from recurrent cytogenetic abnormalities, the role of other rare abnormalities is not well known. Numerical aberrations as the only karyotypic anomalies, including single or multiple losses or gains, are found in approximately 15% of all cytogenetically abnormal hematological malignancies [1]. Among them, isolated trisomy of chromosome 6 is a rare abnormality. It is difficult to determine the prognostic significance of trisomy 6 in acute myeloid leukemia (AML) because of the paucity of clinical publications [2]. Here we present a case of AML with karyotypic abnormality of trisomy 6.

## CASE PRESENTATION

A 21-year-old female reported to the Department of Hematology, All India Institute of Medical Sciences, New Delhi, India, with complaints of generalized weakness and fatigue of 2 weeks in duration. She was found to be pale and had received packed red cell transfusions during this period. On general examination she had anemia, fever, and sternal tenderness. There was no lymphadenopathy or bleeding manifestations. On systemic examination, she had hepatomegaly 6 cm below the costal margin, but the spleen was not palpable.

Complete blood count revealed a hemoglobin level of 5.4 g%, total leucocyte count of 56.3x103/µL, and platelet count of 85x103/µL. Peripheral smear showed 25% blasts of monocytoid morphology. Bone marrow aspirates and touch preparation revealed hypercellular marrow filled with monoblasts and promonocytes. On cytochemistry, blasts were positive for myeloperoxidase, nonspecific esterase, and Sudan Black B and negative for periodic acid-Schiff and acid phosphatase. Bone marrow biopsy showed 100% cellularity with diffuse replacement by blasts.

Immunophenotyping was performed by 6-color flow cytometry using a BD FACSCanto (Becton Dickinson, San Jose, CA, USA). Gated blast populations of cells were positive for CD13, CD33, CD34, HLA-DR, cMPO, CD64, and CD10. Bone marrow morphology, cytochemistry, and immunophenotyping were consistent with the diagnosis of AML-M5.

**Figure 1:**
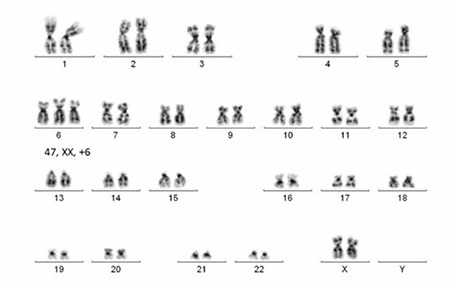
GTG banding 47,XX, +6.

Cytogenetic analysis was performed on a short-term unstimulated bone marrow culture with and without colcemid using standard cytogenetic techniques [3]. G-banded metaphases using trypsin and Giemsa staining were analyzed using an automated karyotyping system (MetaSystems GmbH, Altlußheim, Germany). The karyotype was reported according to the 2009 International System of Human Cytogenetic Nomenclature [4]. Analysis of 20 metaphases showed trisomy 6 in 12 metaphases while the remaining 8 metaphases were normal: 47, XX, +6 (12)/46,XX (8) (Figure 1).

She was started on 7+3 induction with cytarabine at a dose of 100 mg/m2/day as a continuous infusion over 24 h for 7 days and daunorubicin at 60 mg/m2/day as a short infusion for 3 days. After the first induction, bone marrow on day 14 revealed persistence of blasts (34%). Hence, she was given a second induction with mitoxantrone and high-dose cytarabine (HAM). After treatment with HAM, the patient developed septicemia with cardiac dysfunction and bilateral fungal pneumonia, for which she was treated appropriately. After blood counts recovered, bone marrow testing was repeated and she was found to be in remission. She then received 2 more cycles of high-dose cytarabine as postremission therapy. She recovered uneventfully, and is presently being followed up with on a regular basis. Counselling regarding HLA-typing with her sibling and possible need for bone marrow transplantation was given, but this was deferred due to monetary constraints. Informed consent was obtained.

## DISCUSSION AND REVIEW OF THE LITERATURE

Gains and losses of whole chromosomes are frequently found in hematological malignancies, either identified as solitary abnormalities at diagnosis or superimposed on other abnormalities in later stages of the disease [1]. Trisomy 6 as the sole karyotypic abnormality is a rare but recognized finding in hematological disorders. The mechanisms by which trisomies contribute to leukemogenesis are largely unknown, but 2 processes have been suggested. The first is a gene dosage effect as a direct result of the trisomy, with the extra copies of the gene leading to overexpression. The second is underlying cryptic gene rearrangement or mutation of genes on the additional chromosome [5].

A literature search reveals that only 14 cases of AML and 5 cases of myelodysplastic syndrome (MDS) have been reported with isolated trisomy 6. It has been seen in patients with aplastic anemia (AA), MDS, and AML, and is very rarely seen in childhood mixed-lineage leukemia, lymphoblastic transformation of chronic myeloid leukemia, and chronic myeloproliferative disorders. In most of the published cases, patients usually had hypocellular marrow with erythroid dysplasia or they proceeded from AA to AML [6,7]. It has also been reported as an associated finding along with other cytogenetic abnormalities. In contrast to this, our patient presented with a short duration of pallor and fever and was found to have hypercellular bone marrow with replacement of normal marrow elements by blasts. 

In our case, blasts were positive for CD34 and HLA-DR, similar to previous cases, which indicates the primitive nature of the blasts [8]. Response to treatment was also poor as our patient failed to respond to the first induction with persistence of blasts in the marrow at day 14. However, our patient went into remission with the second induction.

Due to the limited number of cases reported in the literature, the role of trisomy 6 as the sole cytogenetic abnormality is not clear, but from the data available, it is likely that this clonal cytogenetic abnormality is associated with the primitive nature of blasts and poor response to treatment [1,9]. More studies are required to ascertain the role of trisomy 6 in the development of leukemia as well as in prognosis.

## CONCLUSIONS

Isolated trisomy 6, although found in many hematological disorders, is rare as a sole abnormality in patients with AML. Although it has been reported more commonly in AA and MDS, it can also be observed in patients with de novo AML. The prognostic significance of this abnormality is not well established at present.
